# Supplementary material for: DeepACSON automated segmentation of white matter in 3D electron microscopy
Source: Commun Biol. 2021 Feb 10;4:179. doi: 10.1038/s42003-021-01699-w (PMC7876004; doi:10.1038/s42003-021-01699-w)
Supplement: Supplementary file 2 — Supplementary Information [file 42003_2021_1699_MOESM2_ESM.pdf]

DeepACSON Automated Segmentation of White Matter in 3D Electron Microscopy

Ali Abdollahzadeh[1], Ilya Belevich[2], Eija Jokitalo[2], Alejandra Sierra[1*+], and Jussi Tohka[1+]

[1]A. I. Virtanen Institute for Molecular Sciences, University of Eastern Finland, Kuopio, Finland

[2]Electron Microscopy Unit, Institute of Biotechnology, University of Helsinki, Helsinki, Finland

[*]alejandra.sierralopez@uef.fi

[+]These authors contributed equally

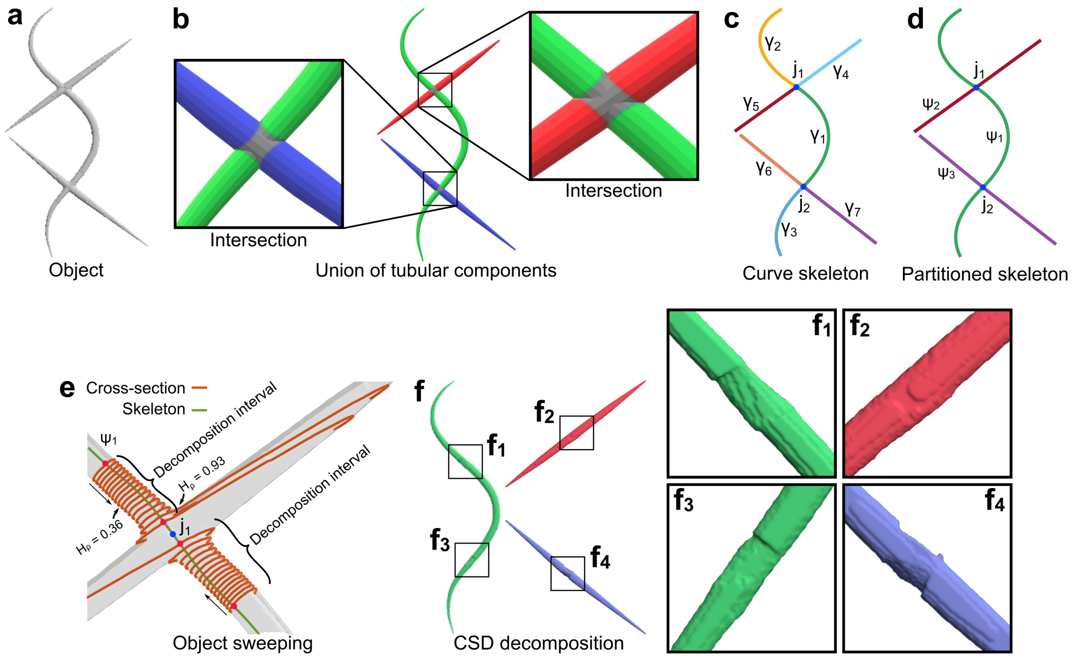

Supplementary Figure S1. Cylindrical shape decomposition algorithm[1]. (**a**) An object is a union of several semantic-components. (**b**) Thesemantic-components of the object in (**a**) are color-coded. Intersections of the semantic-components are colored grey. (**c**)The curve skeleton of the synthetic object in (**a**) is the union of all skeleton branches. Skeleton branches are color-coded
and denoted as γ. We defined a skeleton junction-point j as such a point that skeleton branches connect. Junction-points are shown as blue-filled-circles. (**d**) We found m maximal length sub-skeletons denoted as ψ via minimizing an orientation cost-function. The sub-skeletons are color-coded. (**e**) On a sub-skeleton ψ and in the proximity of a junction-point $j \in \psi$, we defined two decomposition intervals. The boundaries of decomposition intervals are shown with red-filled-circles. In each interval, the cross-section of the object was swept along ψ and towards the joint j to find a critical point. At a critical point, the normalized Hausdorff distance H_ρ between a cross-sectional contour and the mean of visited cross-sectional contours exceeds θ_H. Sweeping directions are shown with arrows. (**f**) We cut the object at critical points to obtain object-parts. The object-parts along the same sub-skeleton were assigned the same label to construct a semantic-component. The semantic-components were further reconstructed between their comprising object-parts using generalized cylinders, magnified in (**f_1-f_4**). The synthetic object in (**a**) comprised of seven object-parts, and our algorithm decomposed it into three semantic-components.

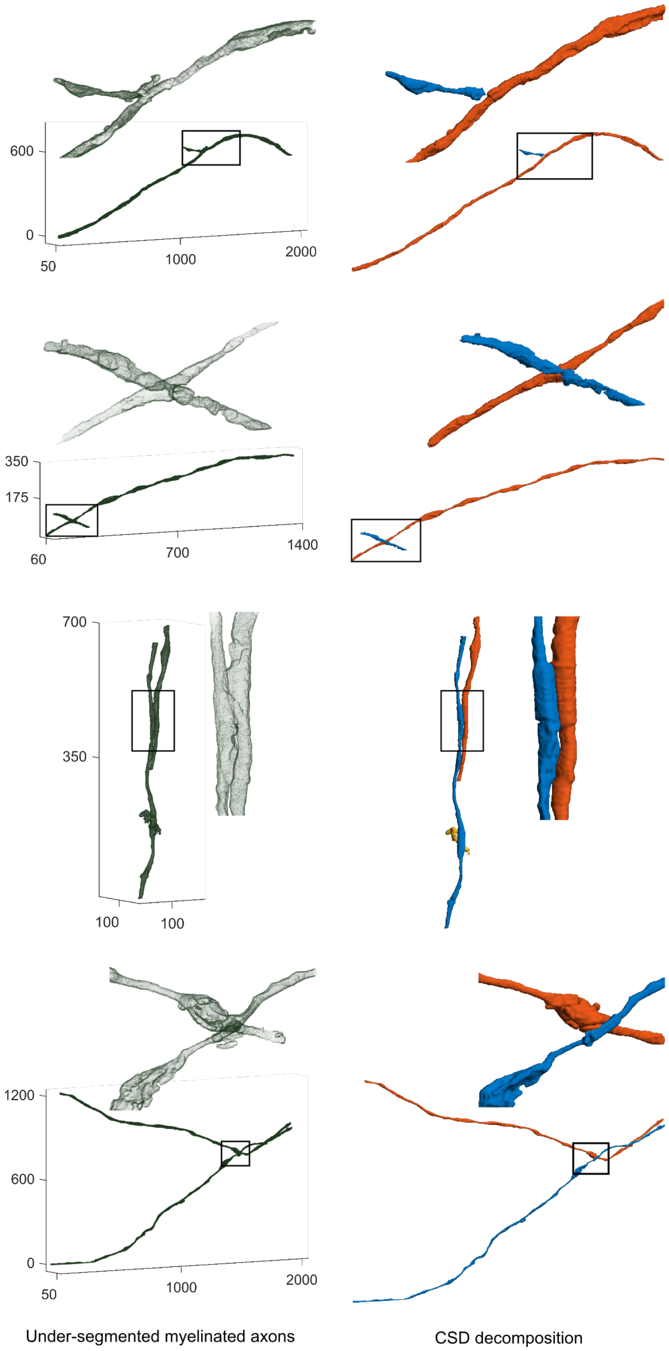

Supplementary Figure S2. Decomposition of Sunder-segmented myelinated axons into their semantic axonal compo nents using the CSDalgorithm[1].

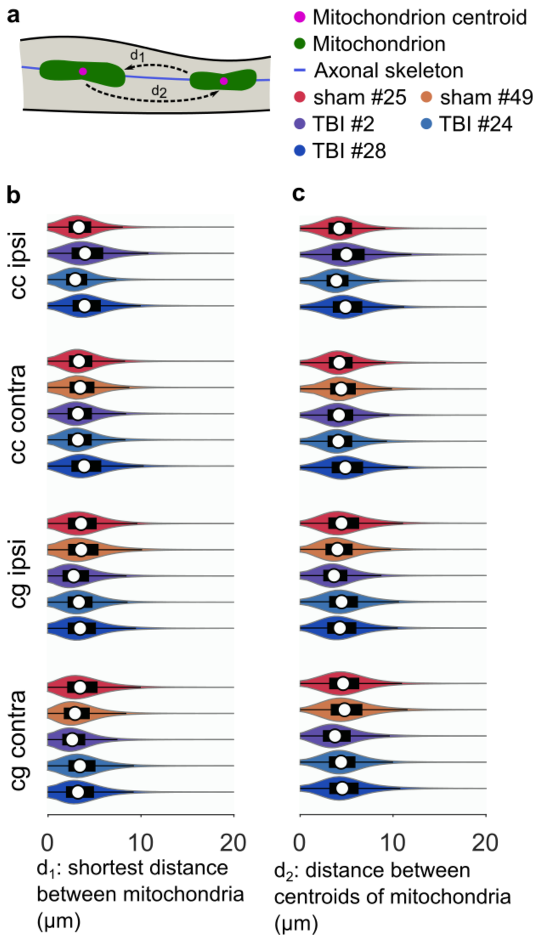

Supplementary Figure S3. Quantification of the inter-mitochondrial distance. (**a**) We defined the inter-mitochondrial dista nce in two, alternateways: 1) we projected the entirety of mitochondria on the axonal skeleton and measured the shortest geode sic distance between

two consecutive mitochondria (d_1); 2) we projected the centroids of mitochondria on the axonal skeleton and measured the geodesic distance between the consecutive projected centroids (d_2). (**b**, **c**) We compared the inter-mitochondrial distance, (**b**: d_1 definition, **c**: d_2 definition), between sham-operated and TBI rats. We did not find significant differences between the groups in any of the brain areas. On each bean plot, the central mark indicates the median, and the left and right edges of the box indicate the 25th and 75th percentiles, respectively. The whiskers extend to the most extreme data points not considered outliers. The colors correspond with the animal ID.

a)

Model		Dataset	Precision	Recall	F1 score
DCNN-mAx	Train set	sham #25 contra	0.908<br>0.925	0.924<br>0.932	0.916<br>0.929
		sham #49 ipsi	0.925<br>0.943	0.882<br>0.958	0.903<br>0.951
		TBI #2 ipsi	0.917<br>0.944	0.938<br>0.920	0.928<br>0.932
		TBI #28 contra	0.899<br>0.912	0.910<br>0.906	0.905<br>0.908
	Test set	sham #25 ipsi	0.931<br>0.946	0.894<br>0.813	0.912<br>0.875
		sham #49 contra	0.746<br>0.910	0.840<br>0.719	0.790<br>0.803
		TBI #2 contra	0.937<br>0.938	0.849<br>0.805	0.891<br>0.866
		TBI #24 contra	0.947<br>0.893	0.882<br>0.895	0.913<br>0.894
		TBI #24 ipsi	0.947<br>0.893	0.898<br>0.897	0.922<br>0.895
		TBI #28 ipsi	0.904<br>0.931	0.877<br>0.751	0.890<br>0.831
DCNN-cN	Train set	sham #25 contra	0.995	0.965	0.980
		sham #49 contra	0.988	0.947	0.967
		sham #49 ipsi	0.994	0.964	0.979
		TBI #2 ipsi	0.989	0.782	0.873
		TBI #24 contra	0.994	0.961	0.977
		TBI #28 contra	0.984	0.966	0.975
SVM (LOGO CV)	Myelinated axons	Sham	0.988	0.988	0.988
		TBI	0.920	0.991	0.955
SVM (LOGO CV)	Cell nuclei	Sham	0.961	0.977	0.969
		TBI	0.964	1	0.981
Final segmentation (expert)	Myelinated axons	Samples from all datasets	0.965±0.027	0.877±0.061	0.918±0.038
	Mitochondria	Samples from all datasets	0.856±0.100	0.804±0.091	0.823±0.070

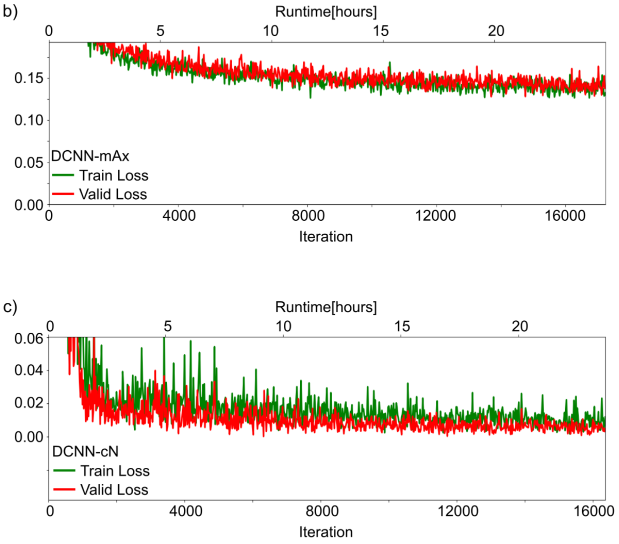

Supplementary Figure S4. DeepACSON evaluation scores. (**a**) We evaluated DeepACSON neural networks on test s ets using Precision,Recall, and F1 score metrics. The same metrics over the training set are provided as a reference. No large differences between

the training and test metrics exist, demonstrating in part that the network did not overfit. In the DCNN-mAx section, red rows show evaluations of myelin semantic segmentation, and gray rows show evaluations of the semantic segmentation of intra-axonal spaces. The DCNN-cN training set included only ten cell nuclei, and we used all the volumes for training. The performance of SVMs was evaluated using leave-one-group-out (LOGO) cross-validation (CV). An expert evaluated the final segmentation of myelinated axons and mitochondria as an added qualitative measure over the entire pipeline. The maximum value of all scores is one. (**b**) The training and validation losses of DCNN-mAx. (**c**) The training and validation losses of DCNN-cN. We trained the networks on an NVIDIA Tesla P100-16 GB GPU for one day.

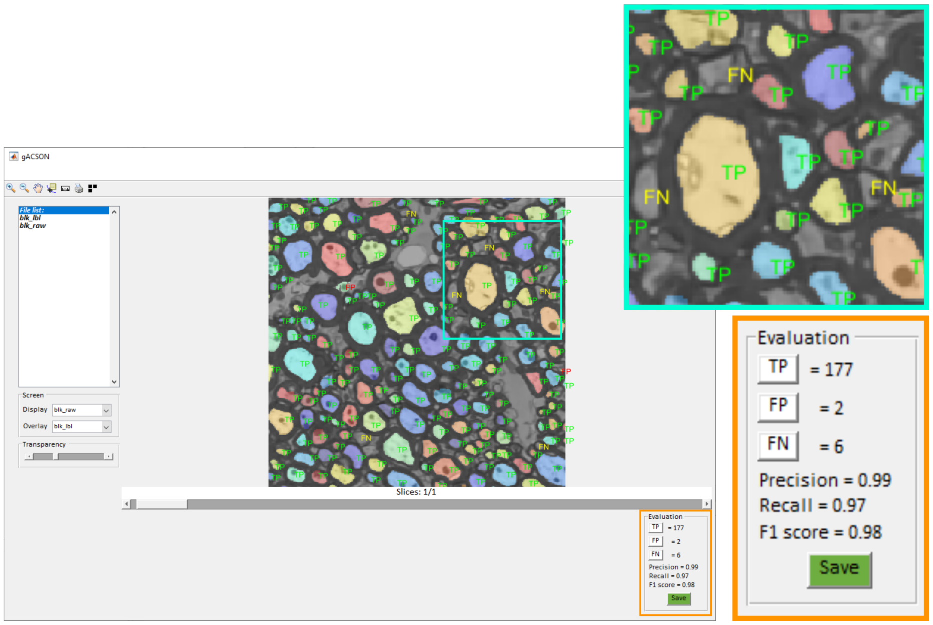

Supplementary Figure S5. DeepACSON evaluation. We developed a GUI-based software tool, gACSON[2], in Matlab to lo ad and visualize thesegmentation for proofreading. gACSON is designed for the visualization of the large-scale image-datasets/ segmentation. Using
gACSON, an expert evaluated the DeepACSON segmentation of myelinated axons and mitochondria at the object-level. We randomly sampled each low-resolution dataset and its corresponding segmentation by non-overlapping images of size 300×300 voxels. The sampled images were quantified for the number of true-positives (TP), false-positives (FP), and false-negatives (FN) to calculate the precision, recall, and F1 score. The expert had no access to the dataset ID nor the sampling location.

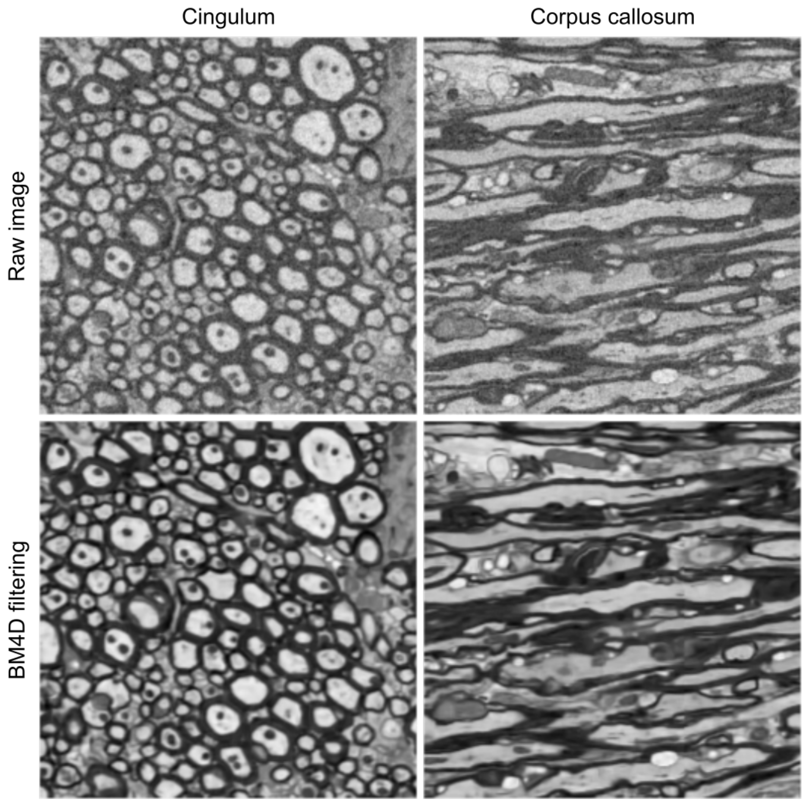

Supplementary Figure S6. BM4D[3] filtering of the low-resolution SBEM datasets. BM4D recognized the noise as Gaussi an-distributed withthe standard deviation in the range [17, 22] in our low-resolution SBEM datasets. Shown images were acquired from the
cingulum and corpus callosum of low-resolution sham #25 dataset.

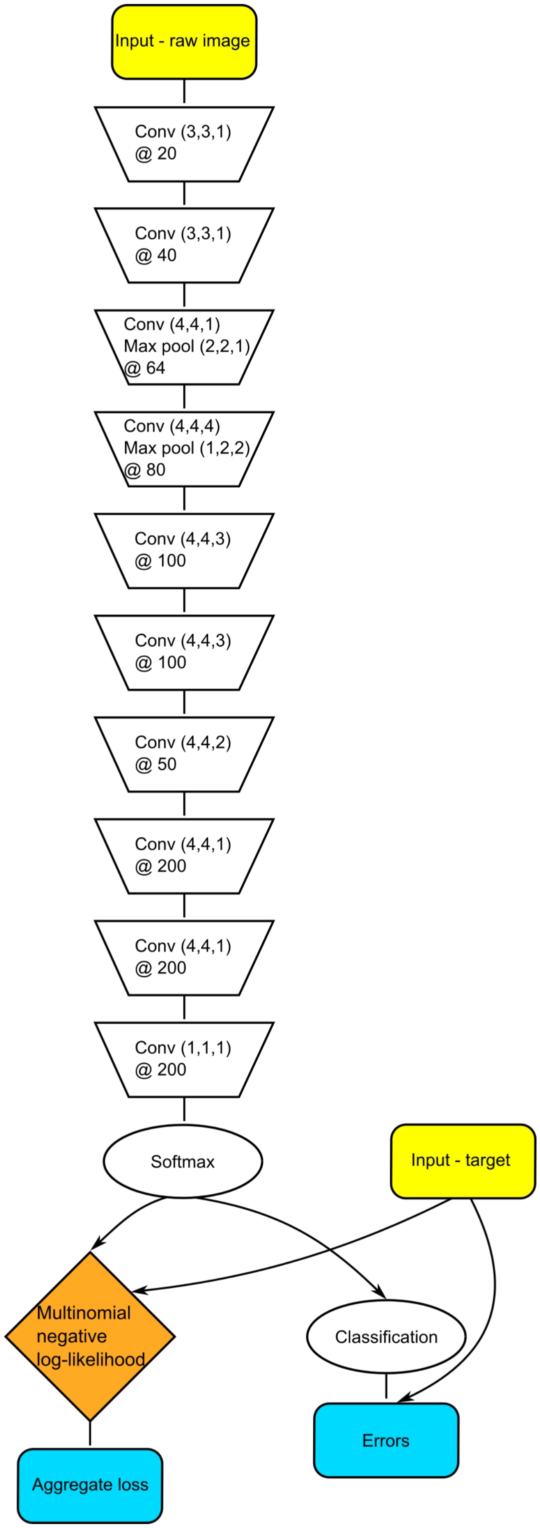

Supplementary Figure S7. The architecture of DCNNs used in DeepACSON. We used the same architecture for DCNN-mAx and DCNN-cN.The size of the convolutional kernels is denoted as $Conv(x,y,z)$. The number of channels/feature maps created from a layer of convolutional kernels is denoted by $@n$. The size of the max pooling operation is denoted as $Max\,pool(x,y,z)$.

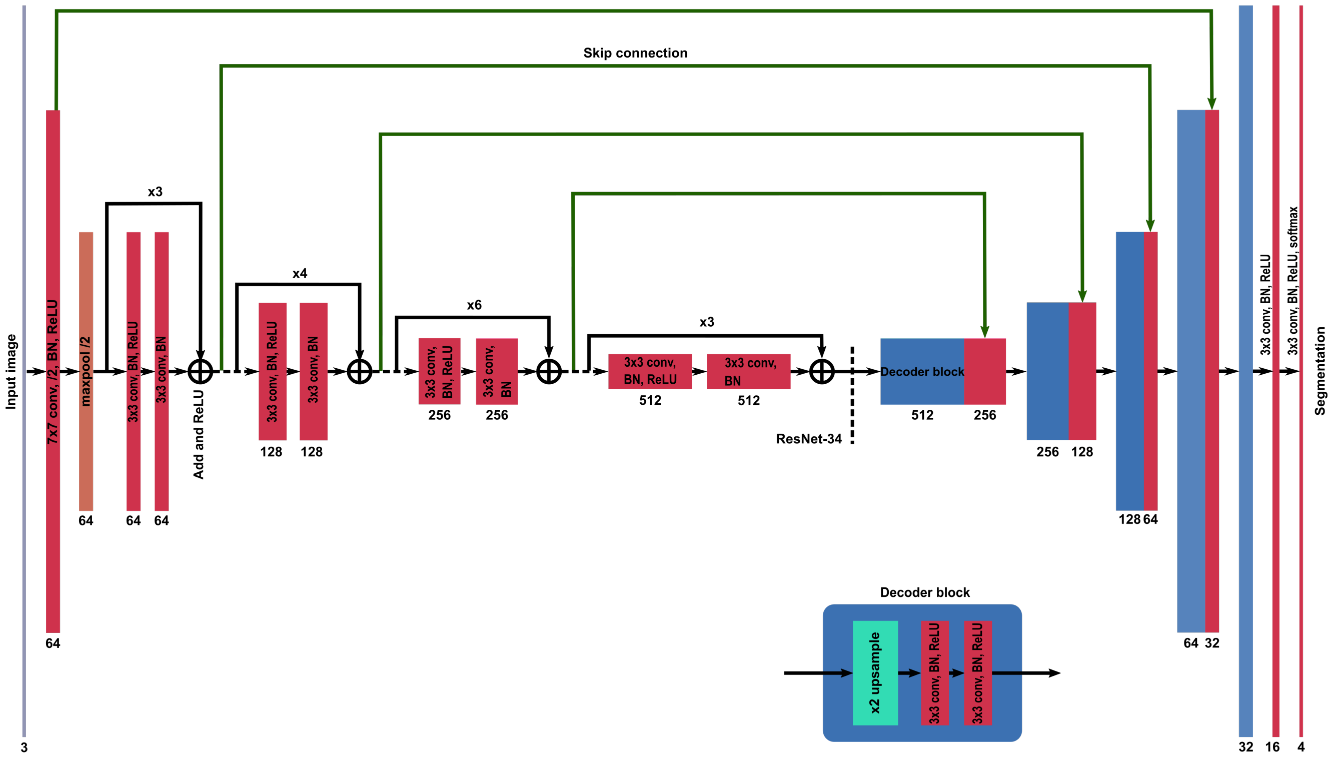

Supplementary Figure S8. A U-Net[4] architecture with residual modules. We used a ResNet-34[5], pre-trained on the Im ageNet dataset[6], asthe encoder of the U-Net. In the encoding path of the U-Net, the height and width of the feature maps were halved, and the

depth of the feature maps was doubled. In the decoding path of the U-Net, the height and width of the feature maps were doubled, and the depth of the feature maps was halved. The basic residual blocks of ResNet-34 were constructed on 3×3 convolutional layers, using rectified linear unit (ReLU) as the activation function, and batch normalization (BN)[7]. The basic decoding blocks applied nearest-neighbor interpolation for up-sampling the feature maps to recover the spatial resolution of input images. Feature maps generated in the encoding path were concatenated to the corresponding feature maps in the decoding path by the skip connections. The model was trained by minimizing cross-entropy loss.

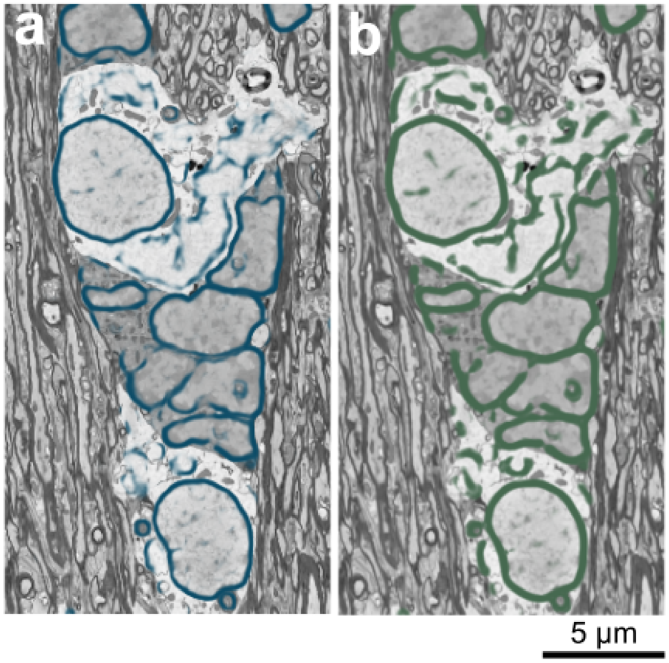

Supplementary Figure S9. Frangi filtering[8] the probability map of the membrane of cell nuclei. (**a**) The probability map of the membrane ofcell nuclei returned by DCNN-cN. (**b**) Application of Frangi filtering on (**a**).

Supplementary Table S1. Characteristics of the low-resolution (LR) and high-resolution (HR) SBEM datasets. We collected the low-resolution images from the ipsi- and contralateral corpus callosum and cingulum for each rat. The low-resolution images from the ipsilateral hemisphere of the sham #49 rat included only the cingulum. The high-resolution images were collected from theipsi- and contralateral corpus callosum. The size of datasets is given in voxels (x,y,z).

Condition	Rat ID	LR-size (voxel)	LR (nm^3)	HR-size (voxel)	HR (nm^3)
Sham	#25 contra	$2044 \times 4096 \times 1306$	$50 \times 50 \times 50$	$1042 \times 1048 \times 285$	$13.8 \times 13.8 \times 50$
	#25 ipsi	$4096 \times 2048 \times 1384$	$50 \times 50 \times 50$	$1049 \times 1076 \times 285$	$15.4 \times 15.4 \times 50$
	#49 contra	$4096 \times 2048 \times 1882$	$50 \times 50 \times 50$	$1081 \times 1053 \times 285$	$18.3 \times 18.3 \times 50$
	#49 ipsi	$2048 \times 2048 \times 1210$	$50 \times 50 \times 50$	$1037 \times 1058 \times 285$	$13.0 \times 13.0 \times 50$
TBI	#2 contra	$4096 \times 2048 \times 1086$	$50 \times 50 \times 50$	$1048 \times 1124 \times 285$	$15.0 \times 15.0 \times 50$
	#2 ipsi	$2154 \times 4134 \times 620$	$50 \times 50 \times 50$	$1343 \times 1316 \times 285$	$15.0 \times 15.0 \times 50$
	#24 contra	$4091 \times 2028 \times 1348$	$50 \times 50 \times 50$	$1289 \times 1280 \times 285$	$15.0 \times 15.0 \times 50$
	#24 ipsi	$2946 \times 2162 \times 1250$	$50 \times 50 \times 50$	$1290 \times 1295 \times 285$	$15.0 \times 15.0 \times 50$
	#28 contra	$4096 \times 2048 \times 1278$	$50 \times 50 \times 50$	$1076 \times 1051 \times 285$	$16.5 \times 16.5 \times 50$
	#28 ipsi	$4075 \times 2000 \times 1300$	$50 \times 50 \times 50$	$1035 \times 1056 \times 285$	$16.5 \times 16.5 \times 50$

Supplementary Table S2. Volumetry of ultrastructures. The volume of myelin and myelinated axons was expressed as a percentage of thecorresponding SBEM dataset. The volume fraction that was occupied by a cell body/process varied between the datasets. Therefore, a direct comparison of the volumes is not reasonable.

Treatment	Tissue	Rat ID	Myelin (%)	mAxons (%)	Cell nuclei	mAxons	Mitochondria
Sham	Cc	#25 contra	48.74	22.24	218	42 318	168 512
		#25 ipsi	49.07	23.17	214	43 209	175 508
		#49 contra	43.53	23.39	161	30 723	116 123
		#49 ipsi	-	-	-	-	-
TBI	Cc	#2 contra	46.42	19.56	133	25 865	91 997
		#2 ipsi	44.13	19.95	124	29 883	58 089
		#24 contra	51.38	19.40	221	49 866	172 241
		#24 ipsi	52.20	22.94	102	23 256	96 032
		#28 contra	42.32	19.31	226	34 804	104 102
		#28 ipsi	40.16	18.61	213	35 553	114 204
Sham	Cg	#25 contra	52.61	29.14	90	16 076	58 583
		#25 ipsi	49.72	28.58	97	15 868	62 838
		#49 contra	50.18	28.19	70	10 537	55 825
		#49 ipsi	48.42	26.49	126	16 932	71 742
TBI	Cg	#2 contra	49.14	22.76	120	23 039	97 777
		#2 ipsi	42.98	12.19	100	14 094	21 722
		#24 contra	49.32	21.98	76	16 495	58 180
		#24 ipsi	41.26	14.72	108	20 374	63 798
		#28 contra	42.68	22.89	131	17 510	77 116
		#28 ipsi	39.79	16.58	167	18 297	48 830

Supplementary Table S3. Computation time. M1: Intel Core i7 6700 CPU 3.4 GHz with 64 GB RAM. M2: 2 $\times$ Intel Xeon E5 2630 CPU2.4 GHz machine with 512 GB RAM. M3: NVIDIA Tesla P100-16 GB GPU. Because the size of datasets and the number of instances segmented in each dataset are different, we measured the computation time based on the sham #25 dataset in the last column.

Process	Machine	Time	Time/dataset (h)
BM4D filtering	M1 - MATLAB R2017b	0.056 MB/s	54.23
Training DCNN-mAx	M3 - Python 2.7	24 h	-
Training DCNN-cN	M3 - Python 2.7	24 h	-
DCNN-mAx inference	M3 - Python 2.7	0.297 MB/s	10.23
DCNN-cN inference	M3 - Python 2.7	0.299 MB/s	10.16
CSD	M2 - Python 2.7	$\sim$ 113.8 s/myelinated axon	44.66
Myelinated Axon feature extraction	M2 - MATLAB R2017b	$\sim$ 15 s/myelinated axon	5.89
2D Frangi filtering	M1 - MATLAB R2017b	0.267 MB/s	11.38
Cell nucleus feature extraction	M1 - MATLAB R2017b	$\sim$ 30 s/nucleus	5.76
SVM Bayesian optimization	M1 - MATLAB R2017b	173 s	-
SVM inference	M1 - MATLAB R2017b	$\sim$ 4.4 μs/component	5.14×10^{-5}

Supplementary References

1. Abdollahzadeh, A., Sierra, A. & Tohka, J. Cylindrical shape decomposition for 3D segmentation of tubular objects. *CoRR* **abs/1911.00571** (2019). URL http://arxiv.org/abs/1911.00571.

2. Behanova, A., Abdollahzadeh, A., Sierra, A. & Tohka, J. gACSON software to visualize, process, and analyze three-dimensional electron microscopy images (2020). URL https://github.com/AndreaBehan/g-ACSON. DOI 10.5281/zenodo.3693563.

3. Maggioni, M., Katkovnik, V., Egiazarian, K. & Foi, A. Nonlocal Transform-Domain Filter for Volumetric Data Denoising and Reconstruction. *IEEE Transactions on Image Process.* **22**, 119–133 (2013). URL http://ieeexplore.ieee.org/document/6253256/. DOI 10.1109/TIP.2012.2210725.

4. Ronneberger, O., Fischer, P. & Brox, T. U-Net: Convolutional Networks for Biomedical Image Segmentation. In Navab, N., Hornegger, J., Wells, W. & Frangi, A. (eds.) *MICCAI 2015*, 234–241 (Springer International Publishing, Cham, 2015). URL http://link.springer.com/10.1007/978-3-319-24574-4_28. DOI 10.1007/978-3-319-24574-4_28.

5. He, K., Zhang, X., Ren, S. & Sun, J. Deep Residual Learning for Image Recognition. In *2016 IEEE Conference on Computer Vision and Pattern Recognition (CVPR)*, vol. 2016-Decem, 770–778 (IEEE, 2016). URL http://ieeexplore.ieee.org/document/7780459/. DOI 10.1109/CVPR.2016.90.

6. Jia Deng *et al.* ImageNet: A large-scale hierarchical image database. In *2009 IEEE Conference on Computer Vision and Pattern Recognition*, 248–255 (IEEE, 2009). URL http://ieeexplore.ieee.org/lpdocs/epic03/wrapper.htm?arnumber=5206848. DOI 10.1109/CVPRW.2009.5206848.

7. Ioffe, S. & Szegedy, C. Batch normalization: Accelerating deep network training by reducing internal covariate shift. *32nd Int. Conf. on Mach. Learn. ICML 2015* **1**, 448–456 (2015).

8. Frangi, A. F., Niessen, W. J., Vincken, K. L. & Viergever, M. A. Multiscale vessel enhancement filtering. In Wells, W., Colchester, A. & Delp, S. (eds.) *Medical Image Computing and Computer-Assisted Intervention — MICCAI'98. MICCAI 1998. Lecture Notes in Computer Science*, 130–137 (Springer, Berlin, Heidelberg, 1998). URL http://link.springer.com/10.1007/BFb0056195. DOI 10.1007/BFb0056195.
