# Supplementary material for: DeepACSON automated segmentation of white matter in 3D electron microscopy
Source: Commun Biol. 2021 Feb 10;4:179. doi: 10.1038/s42003-021-01699-w (PMC7876004; doi:10.1038/s42003-021-01699-w)
Supplement: Supplementary file 3 — Description of Additional Supplementary Files [file 42003_2021_1699_MOESM3_ESM.pdf]

Description of Additional Supplementary Files

File Name: Supplementary Data 1

Description: The source data file of the morphology analysis of myelinated axons (Fig. 5d).

File Name: Supplementary Data 2

Description: The source data file of the inter-mitochondrial distance analysis in myelinated axons (Fig. 5d).

File Name: Supplementary Data 3

Description: The source data file of the myelinated axon density (Fig. 5e) and cell nucleus density (Fig. 5f).

File Name: Supplementary Data 4

Description: The source data file of quantitative evaluations (Fig. 6a-f) and computation times (Fig. 6g).
